# Cathodal Cerebellar tDCS Combined with Visual Feedback Improves Balance Control

**DOI:** 10.1007/s12311-020-01172-0

**Published:** 2020-07-30

**Authors:** Mehran Emadi Andani, Bernardo Villa-Sánchez, Federico Raneri, Silvia Dametto, Michele Tinazzi, Mirta Fiorio

**Affiliations:** Department of Neurosciences, Biomedicine and Movement Sciences, University of Verona, Via Casorati, 43, 37131 Verona, Italy

**Keywords:** Balance control, Posture, Unipedal stance task, tDCS, Cerebellum, Visual feedback

## Abstract

Balance control is essential to maintain a stable body position and to prevent falls. The aim of this study was to determine whether balance control could be improved by using cerebellar transcranial direct current stimulation (tDCS) and visual feedback in a combined approach. A total of 90 healthy volunteers were randomly assigned to six groups defined by the delivery of tDCS (cathodal or anodal or sham) and the provision or not of visual feedback on balance during the acquisition phase. tDCS was delivered over the cerebellar hemisphere ipsilateral to the dominant leg for 20 min at 2 mA during a unipedal stance task. Body sway (i.e., ankle angle and hip position) was measured as an overall maximal unit in anteroposterior and mediolateral direction, together with participant rating of perception of stability, before (baseline), during (acquisition), and after (final) the intervention. We found a reduction in body sway during the acquisition session when visual feedback alone was provided. When the visual feedback was removed (final session), however, body sway increased above baseline. Differently, the reduction in overall maximal body sway was maintained during the final session when the delivery of cathodal tDCS and visual feedback was combined. These findings suggest that cathodal tDCS may support the short-term maintenance of the positive effects of visual feedback on balance and provide the basis for a new approach to optimize balance control, with potential translational implications for the elderly and patients with impaired posture control.

**Electronic supplementary material:**

The online version of this article (10.1007/s12311-020-01172-0) contains supplementary material, which is available to authorized users.

## Introduction

Balance is essential to maintain a stable and upright stance necessary for walking and other daily life activities. Impaired balance control in certain cerebellar disorders results in decreased quality of life and increased risk of falls [1–4]. This informs us about the importance of the cerebellum in controlling balance, as well as about the need to develop training methods that optimize balance control.

For instance, balance training accompanied by visual feedback has been shown to induce improvement of balance control [5, 6] and reduce postural sway [6–8]. Moreover, the elderly take advantage of visual feedback in balance training [9, 10], in which the visual feedback provides useful cues for performing balancing tasks. According to the guidance hypothesis, the provision of visual feedback during the acquisition phase induces more reliance on the visual than on other movement-relevant input, such as vestibular and proprioceptive information [11–13]. When the feedback is removed, the improvement in motor performance disappears.

A different line of research has tackled the possibility to modulate balance control by means of noninvasive brain stimulation, for example with transcranial direct current stimulation (tDCS) applied to the cerebellum. There is some evidence for the beneficial effect of cathodal [14] and anodal cerebellar tDCS on balance [15, 16]. The cerebellum plays an important role in the processing of performance-related feedback and in the visual guidance of movements [17–21]. Because feedback is necessary to optimize balance, it is reasonable to hypothesize that stimulating the cerebellum during concurrent visual feedback on balance may enhance performance.

The aim of our study was to determine whether the combined delivery of visual feedback and cerebellar tDCS would have a more positive effect on balance control than the delivery of the two approaches alone. For this purpose, we delivered anodal or cathodal or sham tDCS to the cerebellum during a challenging unipedal stance task performed with or without concurrent visual feedback in three groups of healthy volunteers.

We hypothesize that visual feedback may provide the cerebellum with a precise signal about the current postural position, thus facilitating learning of balance control through performance monitoring, which is an essential function of the cerebellum [2, 17–21]. We expect that stimulating the cerebellum during this function may help to reinforce the positive effects of visual feedback on a balance task. We cannot make strong predictions about tDCS polarity-specific effects. While evidence suggests that anodal tDCS to the cerebellum results in faster learning in adaptive motor tasks [22, 23], we know that cathodal tDCS of the cerebellum reduces cerebellar-brain inhibition (CBI) [24, 25], which is also reduced after learning (i.e., adaptive learning) [26, 27].

## Methods

### Participants

The sample size was computed using G-Power 3.1 [28], subjected to *F* tests within-between interaction with six groups and three sessions (baseline, acquisition, final). Assuming an anticipated effect size *f* of 0.25, which is considered the medium according to [29], an *α* error probability of 0.05, power (1-β error probability) of 0.95, a correlation among repeated measures of 0.5, and a nonsphericity correction *ε* of 1, the total sample size was set at 72. We recruited more participants to prevent a reduction of power due to potential dropout.

A total of 90 healthy participants (43 females; mean ± standard deviation [SD] 22.6 ± 2.5 years) were recruited from the student population of the University of Verona. Exclusion criterion was a history of neurological or musculoskeletal disorders. The kicking preference [30] showed that 83 were right-footed. Six groups were formed. Table 1 presents the demographic data (age, sex) and body parameters (right foot dominancy, body weight, body height, foot length) of the six groups.

**Table 1:** Demographic data and body measurements of the six study groups. Data are presented as mean ± standard deviation

Group							
	Cath+VF	Anod+VF	Sham+VF	Cath	Anod	Sham	
No. male/no. female	9 M/6F	8 M/7F	7 M/8F	8 M/7F	7 M/8F	8 M/7F	*χ*^2^ = 0.75, df = 5, *p* = 0.980
Age (years)	23.5 ± 2.2	23.6 ± 3.2	24.1 ± 2.6	21.1 ± 1.4	22.3 ± 2.6	21.2 ± 1.6	*F*_(5,84)_ = 4.74, *p* = 0.001
Right foot dominancy	14	13	14	14	14	14	*χ*^2^ = 1.09, df = 5, *p* = 0.955
Body weight (kg)	65.1 ± 11.6	68.6 ± 14.4	69.0 ± 14.5	66.3 ± 10.5	63.4 ± 9.0	68.6 ± 15.3	*F*_(5,84)_ = 0.61, *p* = 0.696
Body height (cm)	172.9 ± 10.9	171.9 ± 8.7	172.1 ± 7.8	173.1 ± 9.8	174.4 ± 11.7	174.1 ± 8.8	*F*_(5,84)_ = 0.28, *p* = 0.924
Foot length (cm)	24.8 ± 2.1	24.0 ± 1.9	24.2 ± 1.9	25.1 ± 1.4	25.0 ± 2.3	25.2 ± 1.9	*F*_(5,84)_ = 1.11, *p* = 0.362

Written informed consent was obtained from all participants before participation in the study. The study was conducted in accordance with the Declaration of Helsinki and approved by the committee for approval of research on humans (CARU) of the University of Verona.

### Unipedal Stance Task

For the unipedal stance task, participants were asked to stand barefoot for 30 s on the dominant leg while maintaining as stable a position as possible and to watch a PC monitor (25 × 44 cm) at eye level 100 cm in front of them. The nondominant leg was kept suspended with the knee flexed, without hip flexion, and the arms relaxed alongside the body. Subjects had to maintain the upright position by controlling their posture on the dominant leg, without moving the nondominant leg and the arms. After standing on the dominant leg for 30 s, the participants were cued by the word “rest” appearing on the PC monitor to stand with both feet on the ground for the next 30 s. The task was repeated in 10 trials for each experimental session (baseline, acquisition, and final, described in detail below). This is a reliable task to evaluate standing balance [31–34]; it is characterized by more postural sway than normal bipedal stance [35] and requires precise coordination to overcome postural modifications [36, 37].

**Fig. 1:**
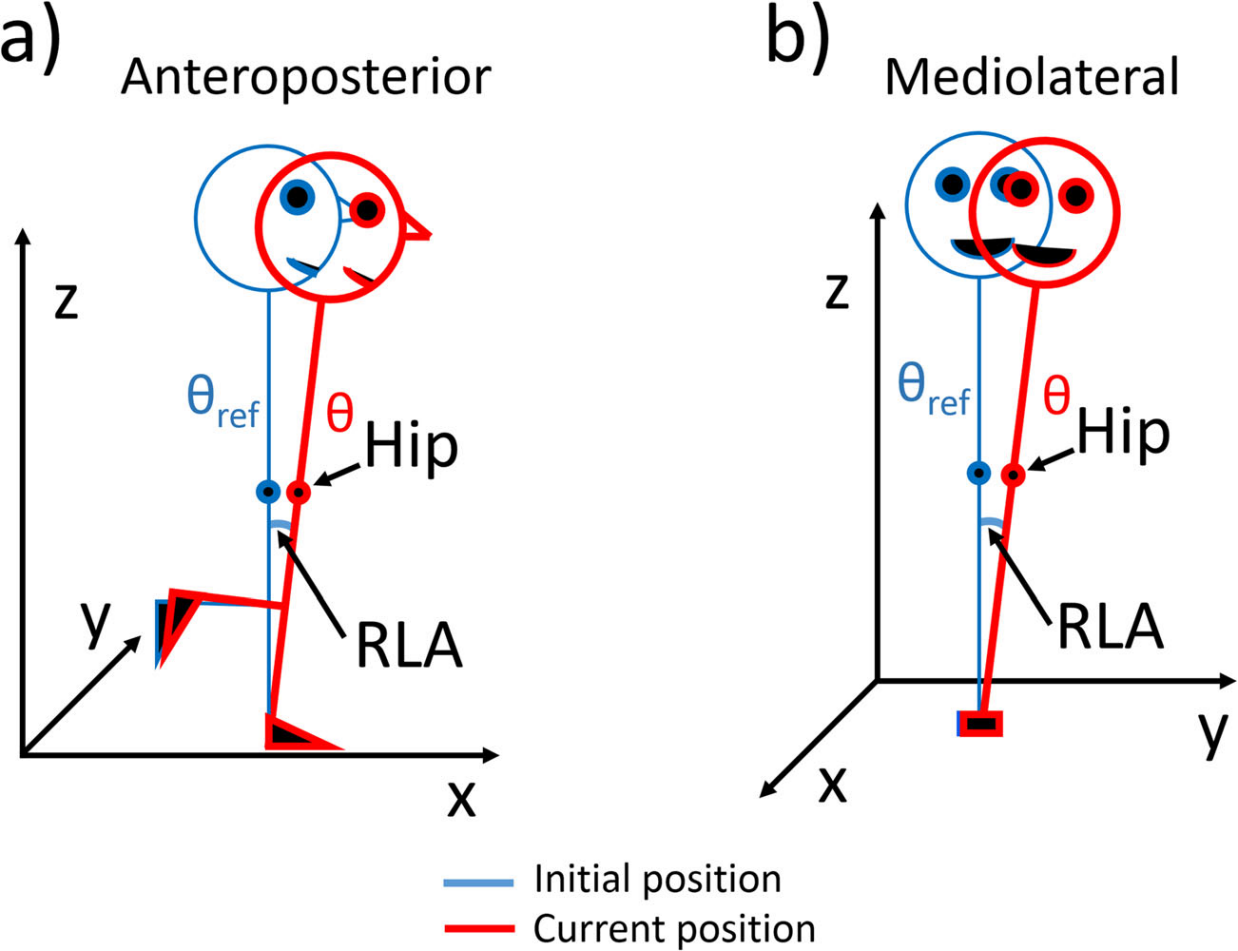
Measures of body sway. The blue line denotes the initial body position measured during the first 3 s of each trial (*θ*_ref_) and the red line denotes the current body position during the trial (*θ*). Body sway was measured as overall maximal displacement in the three-dimensional space (maximal displacement) and as displacement in the anteroposterior direction (**a**) and in the mediolateral direction (**b**). RLA = relative leg angle

Movement during the unipedal stance task was recorded with a custom-made three-dimensional (3D) accelerometer (ADXL345) attached with an elastic band to the front of the thigh of the dominant leg over the rectus femoris muscle. As done in a previous study, we did not position the accelerometer at a fixed distance from the hip but roughly over the thigh [38]. The 3D accelerometer recorded body angular positions (leg angle, *θ*) in the anteroposterior and the mediolateral direction (Fig. 1) [39]. The first 3 s of each trial were used to calculate the initial upright position. This was defined as the initial position of the leg angle (*θ*_ref_) and served as a reference to trace the amount of leg displacement during the rest of the trial. We defined two main parameters of body sway: relative leg angle (RLA) and hip displacement (HD). RLA represents displacement of the ankle angle and was measured as the angular deviation resulting from the difference between the current position of the leg angle (red line in Fig. 1a, b), and the initial position of the leg angle (blue line in Fig. 1a, b) (RLA = *θ*–*θ*_ref_). HD (measured in cm) may reflect displacement of the center of mass, at least in the upright position [40], and was measured as the difference between the current hip position (red line in Fig. 1a, b) and the initial hip position (blue line in Fig. 1a, b). The hip position was determined by measuring the leg angle and the leg length, considering the leg as a rigid link. As described previously, the leg angle was measured with the 3D accelerometer while the leg length was measured manually by the experimenter (i.e., the distance between the hip and the heel). Measuring both RLA and HD could help to discriminate specific effects of tDCS on balance control.

We defined the maximal RLA and the maximal HD as maximal body sway by calculating the absolute maximum peak RLA amplitude and the absolute maximum peak HD amplitude, respectively. To gain a more fine-tuned picture of the body sway changes across different directions, we defined the peak RLA amplitude and the peak HD amplitude also in the anteroposterior and the mediolateral direction (see Supplementary Information for a description and the results of body sway in these directions). Data recorded by the accelerometer were transferred in real-time at a sampling rate of 100 Hz by an Arduino Uno microcontroller onboard a PC for further computation (MATLAB 2014, MathWorks, Natick, MA, USA). The data were low pass filtered by maximally flat zero phase shift digital filtering with a frequency cut at 20 Hz.

### Procedure

**Fig. 2:**
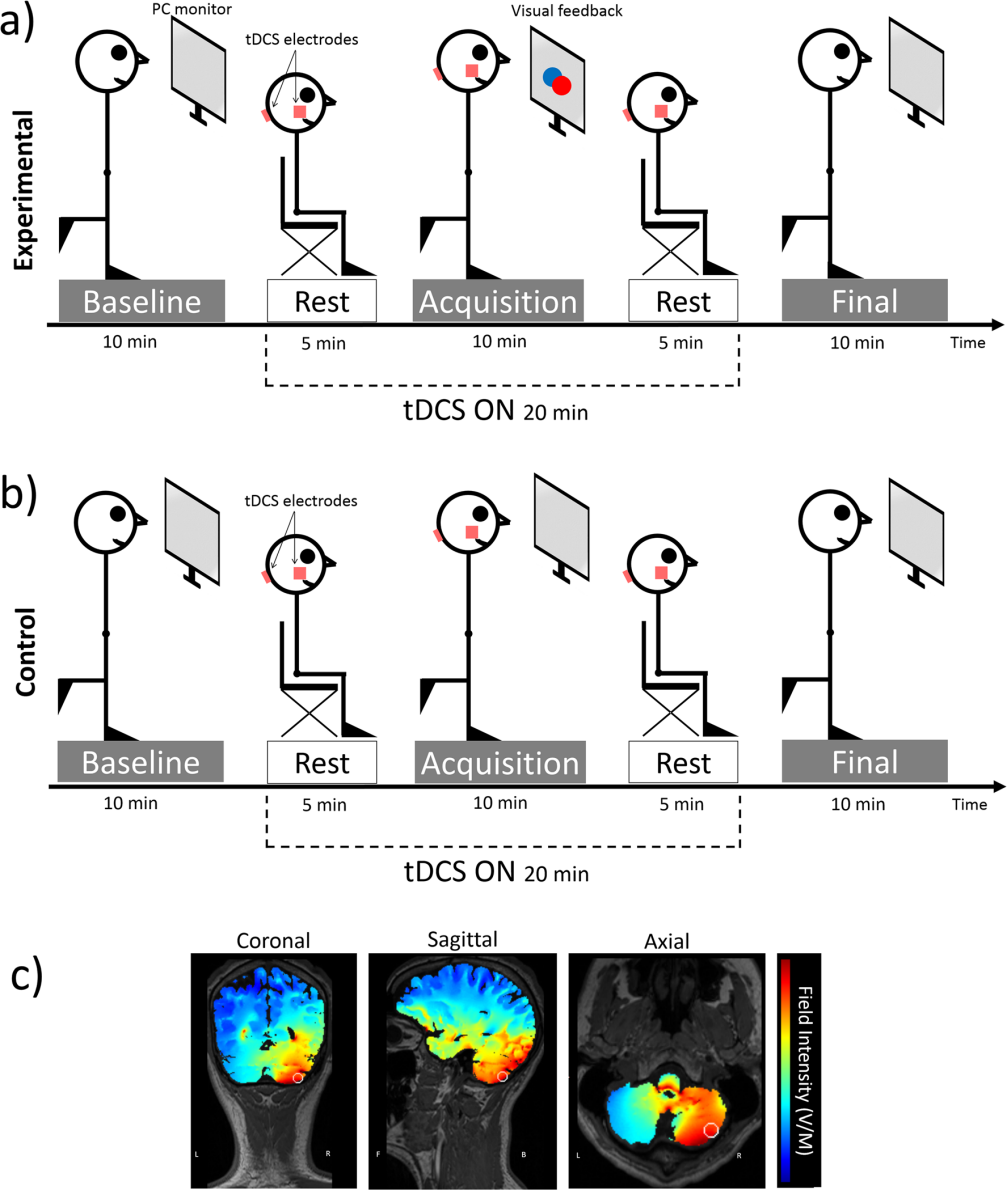
Experimental protocol. The experiment comprised three sessions (baseline, acquisition, final). Participants were asked to perform a unipedal stance task on the dominant leg for 10 trials in each session. Between the sessions, they were allowed to rest for 5 min sitting on a comfortable chair. After baseline acquisition, tDCS electrodes were applied on the cerebellum (reference over the buccinator muscle) and the stimulator was switched on, starting during the 5-min rest period and continuing throughout the acquisition session until the second rest interval (for a total of 20 min). tDCS (cathodal or anodal or sham) was delivered in double-blind fashion. **a** Three experimental groups performed the task while receiving visual feedback on balance during the acquisition session. A blue circle on the PC monitor denoted the initial upright body position and a red circle denoted in real-time the participant’s current body position. Participants were asked to keep the red circle over the blue circle for as long as possible. **b** Three control groups performed the task without receiving visual feedback. **c** Distribution of the electrical field, in the coronal, sagittal, and axial planes, simulated by HD-Explore software. The tDCS montage we used (cerebellar hemisphere and buccinator muscle) allowed us to concentrate the current flow in the stimulated cerebellar hemisphere (the right hemisphere in this figure) with some spread to the vermis. L = left, R = right, F = frontal, B = back. The white circle represents the target location, in our case the cerebellar hemisphere

The experiment started with two familiarization trials (30 s each). The participants performed the unipedal stance task during three sessions: baseline, acquisition, and final (Fig. 2a, b). Each session consisted of 10 trials of executing the unipedal stance task and lasted about 10 min, with 5 min of rest between sessions. After the baseline session, the participants sat while the tDCS electrodes were mounted and tDCS was administered for 5 min before starting the acquisition session. During the acquisition session, the participants performed the unipedal stance task while receiving tDCS (Fig. 2a, b). The participants then rested for 5 min. Finally, for the final session, the electrodes were removed, and the unipedal stance task was repeated.

Three groups of participants (experimental groups: Cath+visual feedback [VF], Anod+VF, Sham+VF) performed the unipedal stance task while receiving visual feedback on balance displayed on the monitor screen and tDCS during the acquisition session (Fig. 2a). The foot position was aligned with the center of the monitor. Hip displacement measured by the accelerometer was linearly converted into real-time displacement displayed as a red circle on the monitor screen. Forward/backward and sideway movements were displayed on the monitor as up/down or left/right movement of the red circle, respectively. The initial hip position computed in the first 3 s of each trial was denoted by a blue circle that remained fixed at the center of the monitor screen during the trial (Fig. 2a). During the first 3 s of each trial, the participants were asked to stand as steady and upright as possible; the blue circle was taken as a reference point of postural stability throughout the trial. During each trial in the acquisition session, the experimental group participants were told to adjust their posture to maintain the mobile red circle over the fixed blue circle for as long as possible.

The other three groups (control groups: Cath, Anod, Sham) served as controls and performed the same task while receiving cerebellar tDCS but without visual feedback during the acquisition session. The PC monitor displayed a plain gray screen during the three sessions (Fig. 2b).

Employing this design, we wanted to evaluate the combined effect of stimulation and visual feedback on balance control (Anod+VF and Cath+VF), the mere effect of visual feedback (Sham+VF), the effect of cerebellar stimulation alone (Anod and Cath), and the effect due to mere repetition of the task (Sham).

Throughout the procedure, we monitored participants’ perception of stability and effort. On completing each session, the participants were asked to rate on a 10-cm visual analog scale (VAS) from 0 (“very unstable”) to 10 (“very stable”) how stable they felt during the task. In addition, after the acquisition and the final session, the participants were asked to rate on a number rating scale (NRS) from − 3 (“much less stable than at baseline”) to 3 (“much more stable than at baseline”), with 0 (the same as at baseline), their perception of stability with respect to the baseline session. The sense of effort was rated on a Borg scale from 0 (rest) to 10 (maximal effort) after each trial in each session [41].

### Transcranial Direct Current Stimulation

Cerebellar transcranial direct current stimulation (tDCS) was delivered through a battery-driven electrical stimulator (DC-Stimulator, BrainStim, E.M.S. Bologna, Italy) with two 5 × 5-cm rubber electrodes inserted in a sponge soaked with saline solution (NaCl concentration 0.9% mM). For anodal stimulation, the anode was placed 3 cm laterally to the inion over the cerebellar hemisphere ipsilateral to the participant’s dominant leg, and the cathode was placed over the ipsilateral buccinator muscle. For cathodal stimulation, the opposite montage was used (i.e., anode over the buccinator muscle and cathode over the cerebellar hemisphere). This montage has proved suitable to stimulate the cerebellum [24, 42, 43]. Simulation of the current flow with HD-Explore software (Soterix Medical, NY, NY, USA) confirmed that the electrical stimulation was mainly localized in the cerebellum (Fig. 2c).

For anodal and cathodal stimulation, a direct current of 2 mA was applied (current density of 0.08 mA/cm^2^) for a total of 20 min with a ramp up/down of 10 s. For sham stimulation, the same intensity of 2 mA was delivered for 30 s at the beginning and 30 s at the end of stimulation (ramp up/down of 10 s) [44]. The 10-s ramp up/down was added to the stimulation time (for both real and sham stimulation). For sham stimulation, the anodal montage was applied in one half of the participants and the cathodal montage in the other half. In this way, both the participants and the experimenter were blind to the type of stimulation. To check whether the participants could recognize the nature of tDCS they had received (or not), at the end of the experiment, we asked them to report whether they thought that tDCS was active or inactive, where the answer “I don’t know” was also accepted. At the end of the experimental procedure, the participants were asked to complete a questionnaire investigating their tDCS sensations and adverse effects [45]. None of the participants reported experiencing adverse effects.

### Data Handling

The raw behavioral data for each participant during each session were inspected to exclude potential outlier values (defined as raw data 2.5 × SD above or below the mean of 10 trials). After having removed the outlier values, we checked for potential outlier subjects, defined as participants whose value for each variable and session was 2.5 × SD above or below the mean of the group.

In each session, we computed the mean of maximal RLA and maximal HD. The mean obtained during the acquisition and final sessions was normalized to the mean obtained at baseline by using Eq. (1) and (2).

1 $$ \mathrm{Index}\%{}_{\mathrm{acquisition}}=\left({\mathrm{Index}}_{\mathrm{acquisition}}-{\mathrm{Index}}_{\mathrm{Baseline}}\right)/{\mathrm{Index}}_{\mathrm{Baseline}}\times 100\% $$

2 $$ \mathrm{Index}\%{}_{\mathrm{final}}=\left({\mathrm{Index}}_{\mathrm{final}}-{\mathrm{Index}}_{\mathrm{Baseline}}\right)/{\mathrm{Index}}_{\mathrm{Baseline}}\times 100\% $$

where index refers to the measures of balance (i.e., maximal RLA and maximal HD).

### Statistical Analysis

Analyses were performed using SPSS software (IBM-SPSS Statistics 21, Armonk, NY, USA). Homogeneity of variances of behavioral and subjective data was checked with Levene’s test, and normality was assessed with the Shapiro-Wilk test.

The mean age of the six groups was analyzed with one-way ANOVA, and post hoc pairwise comparisons were conducted with independent sample *t* test. Gender distribution and right-foot dominancy were analyzed using the chi-squared test. Anatomical parameters (body weight, body height, and foot length) were analyzed using one-way ANOVA with group as the between-subject factor; the *t* test for independent samples was used for post hoc comparisons. The perception of tDCS stimulation was analyzed using the chi-squared test by comparing the frequency of responses “active”, “inactive”, and “do not know” between the groups that received active (Cath+VF, Anod+VF, Cath, Anod) or sham stimulation (Sham+VF, Sham).

Normalized behavioral data were analyzed by means of repeated measures analysis of variance (rmANOVA), with visual feedback (experimental and control) and stimulation (cathodal, anodal, and sham) entered as between-subject factors and session (acquisition, final) as a within-subject factor. Post hoc comparisons were performed using *t* tests for paired or independent samples. Normalized behavioral data were analyzed against zero by means of a one sample *t* test. This analysis allows discerning whether the amount of improvement or worsening in balance control during the acquisition and final sessions was large enough to differentiate it from baseline (the value zero).

Subjective variables (perception of stability, perception of change in stability, sense of effort) were analyzed by means of non-parametric tests. The Kruskal-Wallis test was used to analyze the group (Cath+VF, Anod+VF, Sham+VF, Cath, Anod, Sham) in each session separately. The Mann-Whitney *U* test was used for post hoc pairwise comparisons. The Friedman test was used to analyze the sessions (baseline, acquisition, and final) within each group separately. Post hoc comparisons were performed using the Wilcoxon signed-rank test. The perception of change in stability was analyzed against zero by means of the Wilcoxon signed-rank test to discern whether the amount of improvement or worsening in the subjective perception of stability during the acquisition and final sessions was above or below baseline (represented by the value zero). Bonferroni correction for multiple comparisons was applied where necessary. Effect size between groups was computed with Cohen’s *d* for parametric tests and with *r* for nonparametric tests [29]. The level of significance was set at *p* < 0.050.

## Results

Participants were able to accurately execute the task without touching the floor with the nondominant foot. The groups differed statistically for age (*F*_(5,84)_ = 4.74, *p* = 0.001). Post hoc comparison revealed that the Cath group was younger than the Anod+VF group (*p* = 0.045) and the Sham+VF group (*p* = 0.010); the Sham group was younger than the Sham+VF group (*p* = 0.017) (Table 1). Age was entered as a covariate in the main analysis described below. There was no statistically significant difference in the other demographic and anatomical variables between the groups (Table 1). There was no difference in the frequency of responses “active”, “inactive”, and “do not know” to the tDCS between the active and the sham stimulation groups (for all comparisons, *p* > 0.131, Table 2). Levene’s test revealed homogeneous variance (*p* > 0.174, for all variables) and all data were normality distributed (Shapiro-Wilk test; *p* > 0.062).

**Table 2:** Perception of tDCS. Frequency and % of active, inactive, and do-not- know responses

Groups	Type of response					
	Active		Inactive		Do-not-know	
	No.	%	No.	%	No.	%
Active stimulation (*N* = 60)	30	50	17	28.3	13	21.7
Sham stimulation (*N* = 30)	13	43.3	6	20.0	11	36.7
Comparison between groups	*p* = 0.553		*p* = 0.396		*p* = 0.131	

### Behavioral Data

The overall percentage of outlier values (number of outlier values/total number of trials × 100) and the number of outlier subjects in each group are reported in Supplementary Table 1. Behavioral data are reported as mean ± standard error (SE) (Supplementary Table 2). Here we describe the data for maximal RLA and maximal HD normalized to baseline. Data for RLA and HD obtained in the anteroposterior and the mediolateral direction are reported in the Supplementary Information. For the two indexes (RLA and HD), negative values indicate a reduction in body sway over baseline and therefore better balance control.

**Fig. 3:**
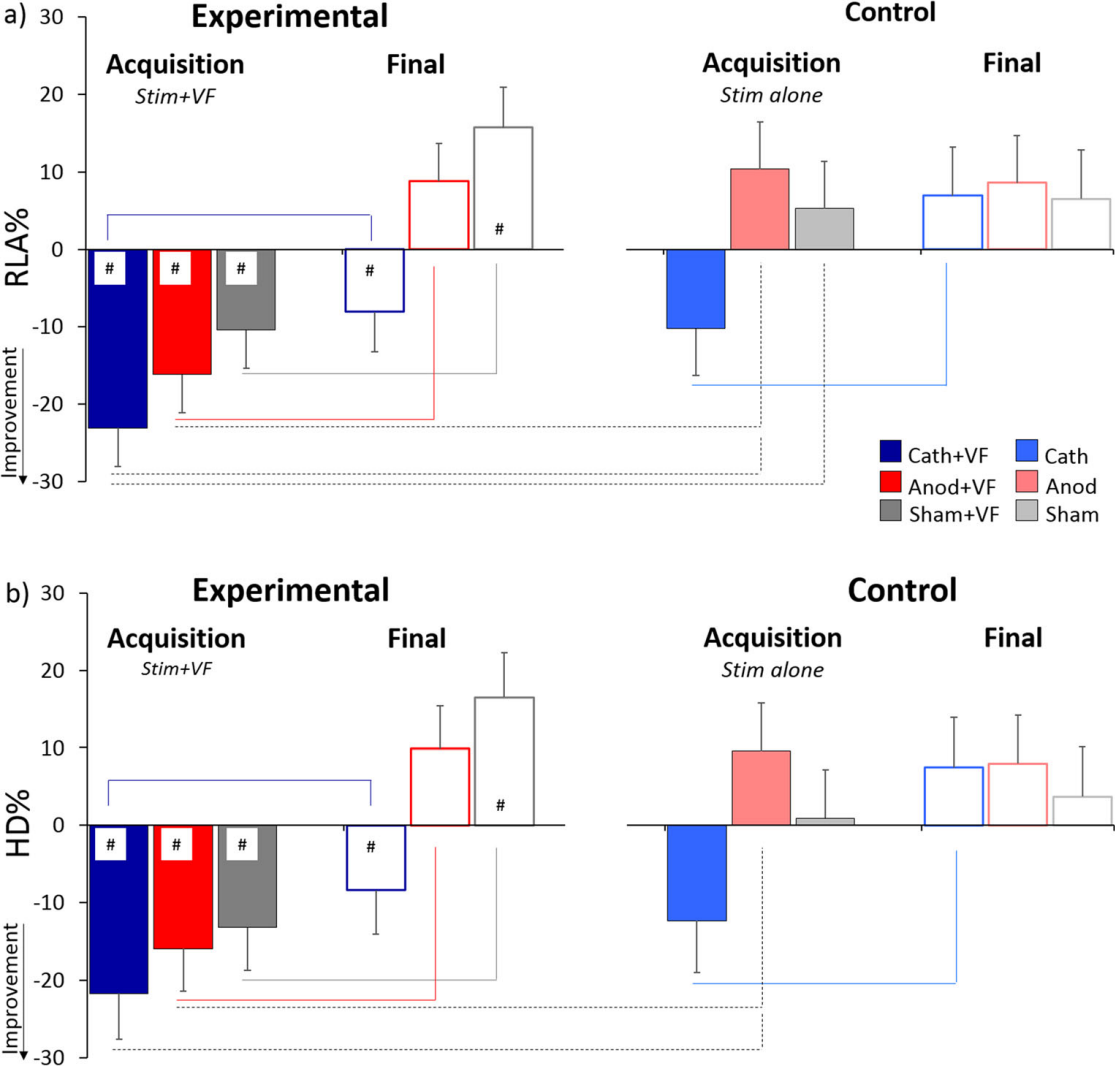
Percentage changes in maximal RLA and maximal HD over baseline. **a** RLA% and **b** HD% were more reduced during the acquisition session (full bars) than during the final session (empty bars) for the experimental groups (left panel) which received visual feedback. The # denotes significant difference from zero. When visual feedback was provided, the values were significantly lower than zero during the acquisition session in the cathodal (blue full bar), anodal (red full bar) and sham (gray full bar) groups. During the final session, the values were higher than zero in the sham group (empty bar with gray border), indicating a drop in performance due to the removal of feedback. The values recorded for the cathodal group (empty bar with blue border) remained lower than zero during the final session, after the removal of visual feedback. In the control groups (right panel) provided no visual feedback, the values for the cathodal group were lower during the acquisition (light blue full bar) than the final session (empty bar with light blue border), indicating that cathodal tDCS per se induced improvement in balance control. Horizontal lines (solid and dashed) indicate significant comparisons between groups. Significance level (*p* < 0.05)

The interaction session × visual feedback was significant for both indexes (RLA: *F*_(1,75)_ = 9.73, *p* = 0.003, η_p_^2^ = 0.115; HD: *F*_(1,73)_ = 9.94, *p* = 0.002, η_p_^2^ = 0.120). Post hoc comparison showed that during the acquisition session, the two indexes were reduced in the groups that received visual feedback compared with the control groups (RLA: *p* < 0.001, Cohen’s *d* = 0.96; HD: *p* = 0.001, *d* = 0.78). This effect was not found for the final session (for both indexes, *p* > 0.709). Moreover, in the groups that received visual feedback, the two indexes were reduced during the acquisition compared with the final session (RLA: *p* < 0.001, *d* = 1.42; HD: *p* < 0.001, *d* = 1.61). No statistically significant difference across sessions was found in the control groups (for both indexes, *p* > 0.115). The significant triple interaction session × stimulation × visual feedback (RLA: *F*_(2,75)_ = 3.24, *p* = 0.045, η_p_^2^ = 0.079; HD: *F*_(2,73)_ = 4.49, *p* = 0.014, η_p_^2^ = 0.110) revealed a reduction in the two indexes in the Cath+VF and Anod+VF groups compared with the Anod group during the acquisition session (post hoc comparison, *p* < 0.023, *d* > 1.30 for all comparisons and indexes) (Fig. 3a, b). In addition, a reduction in RLA was found in the Cath+VF group compared with the Sham group (*p* = 0.002, *d* = 1.62) (Fig. 3a). Finally, the two indexes were reduced during the acquisition session compared to the final session in the Cath+VF, the Anod+VF, Sham+VF, and the Cath group (for all comparisons and indexes, *p* < 0.006, *d* > 1.35) (Fig. 3a, b).

Overall, we also found a reduction in RLA when visual feedback was provided (main effect of visual feedback: *F*_(1,75)_ = 5.86, *p* = 0.018, η_p_^2^ = 0.072) and with cathodal compared with sham stimulation (main effect of stimulation: *F*_(2,75)_ = 4.46, *p* = 0.015, η_p_^2^ = 0.106; post hoc comparison cathodal vs. sham: *p* = 0.024). No other factors were statistically significant (*p* > 0.155).

#### Analysis Against Zero

Values for the Cath+VF group were significantly lower than zero (indicating an improvement in balance control) during both the acquisition (RLA: *p* < 0.001, d = 2.83; HD: *p* < 0.001, *d* = 3.48) and the final session (RLA: *p* = 0.039, *d* = 0.90; HD: *p* = 0.020, d = 1.16) (Fig. 3a, b). The values for the Anod+VF group were significantly lower than zero only during the acquisition session (RLA: *p* = 0.001, *d* = 1.70; HD: *p* = 0.001, *d* = 1.62), while no statistically significant effect was found in the final session (for both indexes, *p* > 0.152) (Fig. 3a, b). The values for the Sham+VF group were significantly different from zero during both the acquisition and the final session but in opposite directions. More precisely, the values were negative (indicating an improvement in balance control) during the acquisition session (RLA: *p* = 0.044, *d* = 0.87; HD: *p* = 0.006, *d* = 1.28) and positive (indicating a reduction in balance control) during the final session (RLA: *p* = 0.027, d = 0.98; HD: *p* = 0.024, *d* = 1.00) (Fig. 3a, b).

### Qualitative Analysis

**Fig. 4:**
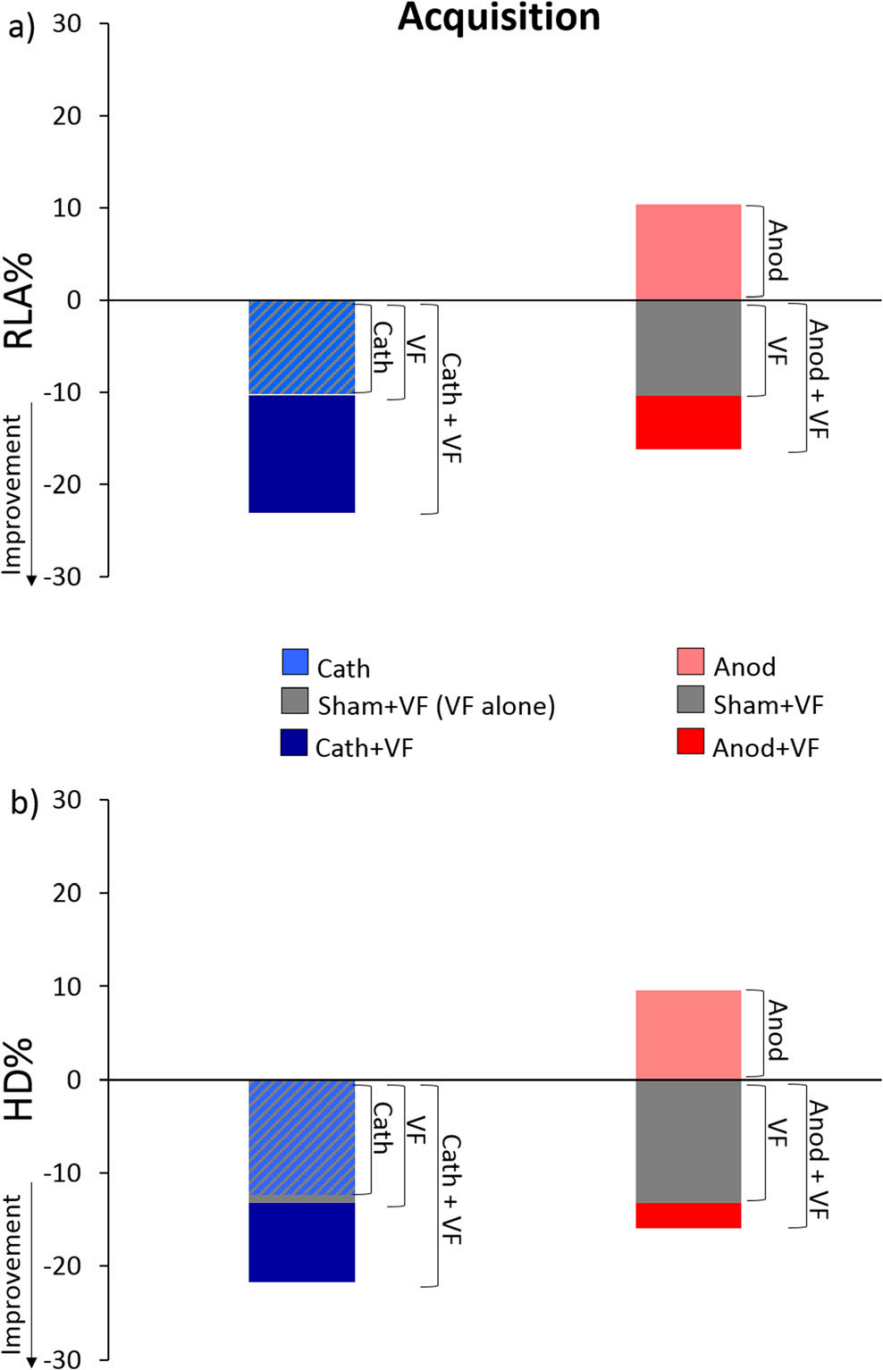
Qualitative superimposition of the percentage change in maximal RLA **a** and maximal HD **b** compared to baseline for the three approaches (stimulation alone, visual feedback alone, stimulation and visual feedback combined) during the acquisition phase

In order to qualitatively appreciate the effects of the interventions separately and combined during the acquisition phase, we superimposed the effects of stimulation alone (Cath and Anod) over the effects of visual feedback alone (Sham+VF) and the effects of the combined approaches (Cath+VF, Anod+VF). We applied this approach to analyze maximal body sway for the two indexes (RLA and HD), which are most indicative of overall balance control. Delivery of cathodal tDCS alone appeared to have an effect comparable to visual feedback alone, whereas combination of the two induced a more robust improvement in balance (Fig. 4a, b). While balance appeared reduced with delivery of anodal tDCS alone, the provision of visual feedback had positive effects (Fig. 4a, b). Moreover, the additional effect of combining the two approaches appeared to be smaller than with cathodal tDCS (Fig. 4a, b).

### Subjective Data

**Fig. 5:**
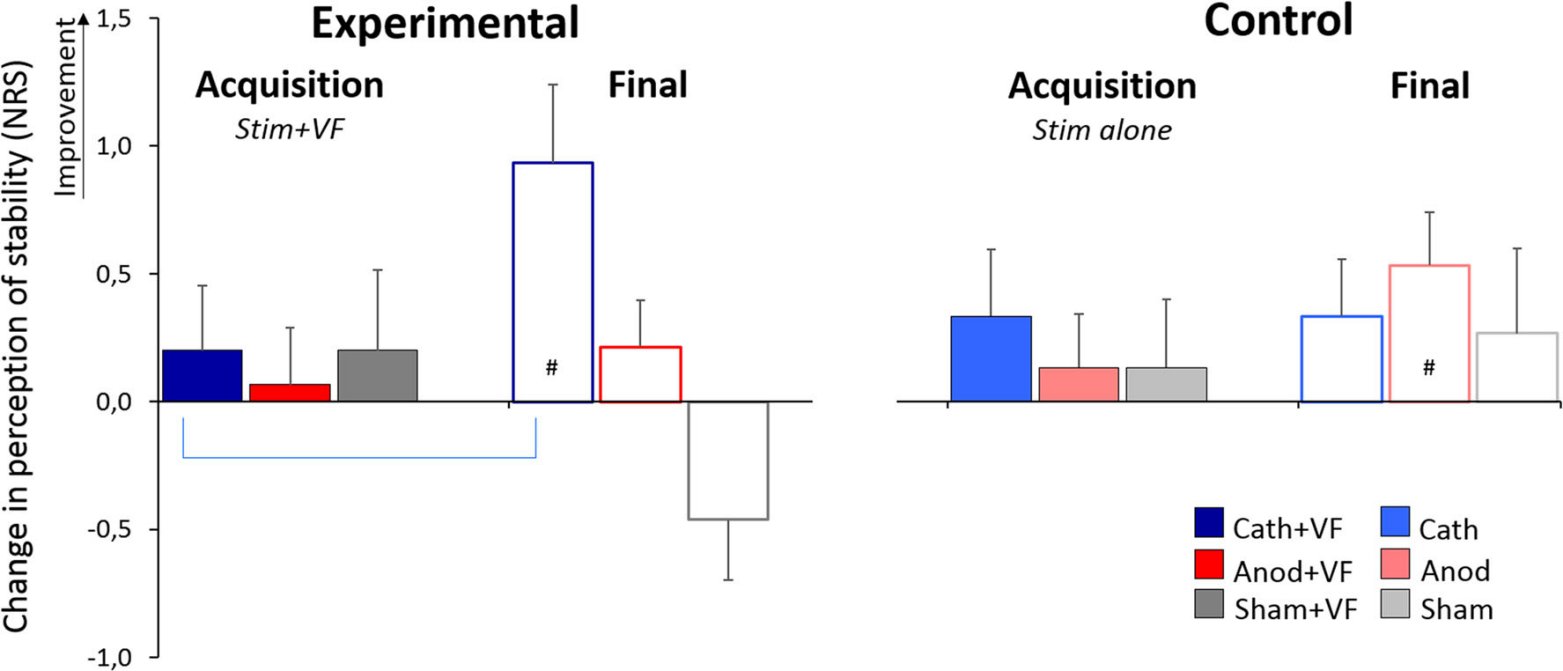
Perception of change in stability. When visual feedback was provided, cathodal tDCS induced a perception of greater stability during the final (blue empty bars) than during the acquisition session (blue solid bars). # denotes significant difference from zero. Significance level (*p* < 0.05)

For the sake of clarity, subjective data are reported as mean ± standard error (SE). The perception of stability rated on the VAS (0 = “very unstable” to 10 = “very stable”) revealed no significant effect (*p* > 0.140). Conversely, the perception of a change in stability rated on the NRS (− 3 = “much less stable than at baseline” to 3 = “much more stable than at baseline”) revealed higher scores for the Cath+VF group during the final (0.93 ± 0.31) compared with the acquisition session (0.20 ± 0.25; Wilcoxon test, *Z* = − 2.48, *p* = 0.013, *r* = 0.45) (Fig. 5). Furthermore, during the final session, the scores were significantly higher than zero for the Anod (0.53 ± 0.21; *Z* = − 2.14, *p* = 0.033, *r* = 0.39) and the Cath+VF group (0.93 ± 0.31; *Z* = − 2.45, *p* = 0.014, *r* = 0.45). No significant effects were found for the other groups (*p* > 0.084).

Analysis of the sense of effort revealed that group was significant only during the baseline session (*χ*^2^ = 18.21, df = 5, *p* = 0.003). Post-hoc analysis revealed that the values for the sham group (2.64 ± 0.27) were significantly higher than those for the Cath+VF (1.52 ± 0.17; U = 41, *p* = 0.044, *r* = 0.54) and the Sham+VF group (1.45 ± 0.19; *U* = 38, *p* = 0.028, *r* = 0.57). No other effect or comparison was significant (*p* > 0.068).

## Discussion

With the present study, we investigated the effects of combined cerebellar tDCS and visual feedback on balance control. Our findings can be summarized as follows: (i) during the acquisition phase, provision of visual feedback per se improved balance control, but this improvement disappeared after the visual feedback was removed; (ii) cathodal tDCS per se induced better balance control during the acquisition phase than during the final phase; (iii) cathodal tDCS combined with provision of visual feedback induced a more robust improvement in balance control than the two approaches alone and this improvement was maintained in the final phase, when the visual feedback and the stimulation were removed. These findings are discussed in relation to the role of visual feedback in influencing balance and the role of the cerebellum in the visual guidance of movement and balance control.

### The Effect of Visual Feedback on Balance Control

For this study, we used an abstract symbol as visual feedback (a circle) to indicate body position. We found that, during the acquisition phase, provision of visual feedback per se had a positive effect on balance, as seen for the Sham+VF group. This finding shared by previous studies that showed that visual feedback of posture in a quiet standing position influences postural sway in diverse populations including healthy, young participants [46], the elderly [9, 10], and patients with Parkinson’s disease [47]. Our paradigm can be considered suitable to induce visually guided enhancement of balance control.

When visual feedback was removed, however, performance dropped to below baseline. This pattern can be explained by the guidance hypothesis [11–13] which posits that when guided by visual feedback individuals learn to visually control their movements and ignore internal signals (i.e., vestibular and proprioceptive) that are also present during execution of movement. This results in dependency on the visual feedback during the acquisition phase so that motor performance worsens when the feedback is removed. Nonetheless, visual feedback on performance is a widely used strategy in the early stages of skill acquisition as it boosts motor learning [13, 48, 49] and helps in generalizing a practiced motor task [50]. Moreover, for this study, we adopted visual feedback under the assumption that because the cerebellum is involved in performance-related feedback and in the visual guidance of movements [17–21], it could play a preferential role when visual feedback on balance is provided.

### The Effects of Cerebellar Cathodal tDCS Combined with Visual Feedback on Balance

The main and novel finding of our study is that a more robust improvement of balance control was observed during the acquisition session when cathodal tDCS was delivered in combination with visual feedback than when the two approaches were delivered separately. The improvement was maintained into the final session when no visual feedback was provided and tDCS was switched off; this hints at short-term retention of newly acquired balance control.

As concerns the acquisition session, we suggest that the visual feedback may have provided the cerebellum with a precise signal about the current postural position, thus facilitating learning of balance control through performance monitoring, which is an essential function of the cerebellum [2, 17–21]. Qualitative superimposition of the diverse approaches shows similar positive effects on body sway for cathodal tDCS alone and visual feedback alone. When cathodal tDCS was combined with visual feedback, body sway was almost twofold less than when the two approaches were delivered alone, hinting at a more robust effect. Also, an improvement in balance was observed after anodal tDCS combined with visual feedback, but the gain in performance was as high as that obtained with cathodal tDCS alone. Whether this difference is related to the polarity-specific effects of cerebellar tDCS on CBI [22] is difficult to know. Future studies may help to clarify this issue by evaluating CBI in our protocol, as previously done for other motor learning tasks [27, 51].

We noted that body sway remained small during the final session in participants who received combined visual feedback and cathodal tDCS (i.e., Cath+VF group) during the acquisition phase. Removal of visual feedback did not result in a drop in performance, as the guidance hypothesis would predict. This finding seems to suggest that the Cath+VF group participants did not develop a dependence on the visual feedback during the acquisition phase so that they maintained good balance control also in the final session. A similar observation was reported by a study that showed that when visual feedback is weak because of low visual contrast, for instance, performance is better on retention tests than after full visual feedback or the absence of visual feedback [52]. This means that while visual feedback is important for good motor performance, the acquired skill is maintained better if performance does not depend on visual feedback.

We cannot exclude that the tDCS current could have spread to the occipital cortex, since we did not directly measure the current flow (but only indirectly simulated it) and since we did not check the participants’ eyesight. It could be argued that if the tDCS current reached the occipital cortex, it could have affected visual acuity, thus reducing the dependency of performance on visual feedback. Neurophysiological studies have shown, however, that cerebellar tDCS does not affect the activity of the visual cortex [22, 53]. A TMS study demonstrated that cerebellar tDCS did not change the excitability of the visual cortex, as measured by changes in visual phosphenes induced by the TMS pulse on the visual cortex [22]. Furthermore, an EEG study demonstrated that cerebellar tDCS did not change the visual evoked potentials recorded from the inion [53]. Hence, taking this evidence together, we think that the effects we found are more likely due to specific stimulation of the cerebellum rather than of the occipital cortex.

A possible, yet speculative, explanation for our finding is that the visual feedback provided essential visual information to guide and optimize balance control while, thanks to cathodal tDCS over the cerebellum, it did not affect the correct processing of other movement–related sensory information (e.g., vestibular and proprioceptive) presented concomitantly with the visual feedback. In other words, cerebellar cathodal tDCS may have allowed the processing of both the visual feedback and other sensory signals, thus facilitating the integration of movement-related sensory information important to develop an optimal motor program and to maintain the acquired skill after the removal of visual feedback. Due to the short duration of the final session (10 min), the current findings can only suggest a short-term maintenance of improved balance control. The precise mechanisms underlying our findings, as well as potential long-lasting effects of the combined approach, should be a future area of focus for other studies.

## Conclusion

Cathodal tDCS of the cerebellum with provision of visual feedback of balance induced better balance control when combined than when the two approaches were delivered separately. Moreover, this improvement persisted after the removal of visual feedback, hinting at short-term maintenance of newly acquired balance control. This evidence may inform interventional approaches designed to optimize balance control in the elderly and in patients with balance disturbances, such as Parkinson’s disease and after stroke. Further studies are needed to define the long-term effects and the precise mechanisms underlying the combined approach used in this study.

## Electronic supplementary material

ESM 1 (DOCX 266 kb)
