# Supplementary material for: Cathodal Cerebellar tDCS Combined with Visual Feedback Improves Balance Control
Source: Cerebellum. 2020 Jul 30;19(6):812–23. doi: 10.1007/s12311-020-01172-0 (PMC7588368; doi:10.1007/s12311-020-01172-0)
Supplement: Supplementary file 1 — (DOCX 266 kb) [file 12311_2020_1172_MOESM1_ESM.docx]

**SUPPLEMENTARY MATERIAL**

**Cathodal cerebellar tDCS combined with visual feedback improves balance control**

Mehran Emadi Andani, Bernardo Villa-Sánchez, Federico Raneri, Silvia Dametto, Michele Tinazzi, Mirta Fiorio*

Department of Neurosciences, Biomedicine and Movement Sciences, University of Verona, Verona, Italy.

**METHODS**

**Body sways in the anteroposterior and the mediolateral direction**

To gain a more fine-tuned picture of body sway changes in different directions, we defined the *peak* RLA amplitude and the *peak* HD amplitude in the anteroposterior and in the mediolateral direction (see Figure 1). Data of the acquisition and final sessions were normalized to baseline by using the same formula described in the main text. Body sway in the anteroposterior and the mediolateral direction represents different components of postural stability [1]. While balance in the anteroposterior direction is under ankle control, displacement in the mediolateral direction is under hip control [2-4]. Hence, dissecting the body sway in the anteroposterior and the mediolateral direction could give further information about the postural strategy the participants used.

**Statistical analysis**

Normalized behavioral data of RLA and HD in the anteroposterior and mediolateral directions were analyzed by means of repeated measures analysis of variance (rmANOVA), with Visual feedback (experimental and control) and Stimulation (cathodal, anodal and sham) entered as between-subject factors and Session (acquisition, final) as a within-subject factor. Post-hoc comparisons were performed using t-tests for paired or independent samples. Normalized behavioral data were analyzed against zero by means of a one sample t-test. This analysis allows to discern whether the amount of improvement or worsening in balance control during the acquisition and final sessions was large enough to differentiate it from baseline (the value zero). Bonferroni correction for multiple comparisons was applied where necessary. Effect size between groups was computed with Cohen’s d for parametric tests and with r for non-parametric tests [5]. The level of significance was set at p < 0.050.

**RESULTS**

Behavioral data are reported as mean ± standard error (SE) (Supplementary Table 2). For the two indexes (RLA and HD), negative values indicate a reduction in body sway over baseline and therefore better balance control.

**Body sway in the anteroposterior direction**

The interaction Session × Visual Feedback was significant (RLA: F_(1,75)_ = 19.01, p < 0.001, η_p_^2^ = 0.202; HD: F_(1,75)_ = 15.49, p < 0.001, η_p_^2^ = 0.171). Post-hoc comparison showed that during the acquisition session the two indexes were reduced in the groups that received visual feedback compared to the control groups (RLA: p < 0.001, d = 1.30; HD: p < 0.001, d = 1.08). This effect was not found during the final session (for both indexes, p > 0.888). Moreover, in the groups that received visual feedback the two indexes were reduced during the acquisition session compared to the final session (RLA: p < 0.001, d = 1.93; HD: p < 0.001, d = 1.92). No statistically significant difference across sessions was found in the control groups (for both indexes, p > 0.254). The significant triple interaction Session × Stimulation × Visual Feedback (RLA: F_(2,75)_ = 4.22, p = 0.018, η_p_^2^ = 0.101; HD: F_(2,75)_ = 5.00, p = 0.009, η_p_^2^ = 0.118) revealed that during the acquisition session the two indexes were reduced in the Cath+VF and the Anod+VF group compared to the Anod and the Sham group (post-hoc comparison, p < 0.036, d > 1.15 for all comparisons and indexes) (Figure S1A, B). In addition, during the acquisition session the RLA index was reduced in the Sham+VF compared to the Anod group (p = 0.016, d = 1.58) and in the Anod+VF group compared to the Cath group (p = 0.025, d = 1.17) (Figure S1A). Finally, the two indexes were reduced in the Cath, the Cath+VF, the Anod+VF, and the Sham+VF group during acquisition compared to the final session (for all comparisons and indexes, p < 0.016, d > 1.09) (Figure S1A, B). Overall, we found that the RLA index was reduced when visual feedback was provided (main effect of Visual Feedback: F_(1,75)_ = 5.49, p = 0.022, η_p_^2^ = 0.068). No other factors were statistically significant (p > 0.377).

*Analysis against zero*. During the acquisition session, the two indexes were significantly lower than zero for the Cath+VF group (RLA: p < 0.001, d = 2.80; HD: p < 0.001, d = 3.23), the Anod+VF group (RLA: p < 0.001, d = 2.94; HD: p < 0.001, d = 2.13), and the Sham+VF group (RLA: p < 0.001, d = 1.90; HD: p < 0.001, d = 1.48). No significant effect was found during the acquisition session for the control groups (p > 0.076). During the final session, no significant effect was found (for both indexes and all groups, p > 0.063).

**Body sway in the mediolateral direction**

Analysis of the two indexes in the mediolateral revealed no significant factors or interactions (for both indexes, p > 0.060) (Figure S2A, B).

*Analysis against zero*. The two indexes were significantly lower than zero for the Cath+VF group during the acquisition (RLA: p = 0.001, d = 1.61; HD: p < 0.001, d = 2.04) and the final session (RLA: p = 0.031, d = 0.91; HD: p = 0.017, d = 1.02) (Figure S2A, B). The RLA index for the Anod+VF group differed from zero only during the acquisition session (: p = 0.046, d = 0.83) (Figure S2A). No effect was found for the other groups (for all comparisons: p > 0.077).

**DISCUSSION**

We found that body sway in the anteroposterior direction was less during the acquisition than during the final session for the experimental groups provided with visual feedback. Moreover, when visual feedback was provided, the values were significantly lower than zero during the acquisition session. These findings suggest that the control of body sway in the anteroposterior direction takes advantage from the presence of visual feedback, independent of the type of tDCS. With regard to the mediolateral direction, we found that the values for the cathodal group provided with visual feedback were lower than zero not only during the acquisition session but also during the final session, after the removal of visual feedback, hinting at a short-term retention of balance control in the mediolateral direction. Of note, body sway in the mediolateral direction was associated with postural instability, with a consequent risk of falls [6]. Hence, finding less mediolateral sway in the group of participants who underwent cathodal ctDCS with visual feedback hints at a potential future translational impact on this approach to persons at risk of falls, like the elderly, patients with Parkinson’s disease or with stroke.

**REFERENCES**

1. Mochizuki L, Duarte M, Amadio AC, Zatsiorsky VM, Latash ML. Changes in postural sway and its fractions in conditions of postural instability. J Appl Biomech. 2006;22(1):51-60. doi: 10.1123/jab.22.1.51.

2. Winter DA, Prince F, Frank JS, Powell C, Zabjek KF. Unified theory regarding A/P and M/L balance in quiet stance. J Neurophysiol. 1996;75(6):2334-2343. doi:10.1152/jn.1996.75.6.2334.

3. Allet L, Kim H, Ashton-Miller J, De Mott T, Richardson JK. Frontal plane hip and ankle sensorimotor function, not age, predicts unipedal stance time. Muscle Nerve. 2012;45(4):578-585. doi:10.1002/mus.

4. Freyler K, Gollhofer A, Colin R, Bruderlin U, Ritzmann R. Reactive balance control in response to perturbation in unilateral stance: interaction effects of direction, displacement and velocity on compensatory neuromuscular and kinematic responses. PLoS One. 2015;10(12):e0144529. doi:10.1371/journal.pone.0144529.

5. Fritz CO, Morris PE, Richler JJ. Effect Size Estimates: Current Use, Calculations, and Interpretation. Journal of Experimental Psychology: General. 2012;141(1):2–18. doi:[10.1037/a0024338](https://www.researchgate.net/deref/http%3A%2F%2Fdx.doi.org%2F10.1037%2Fa0024338?_sg%5B0%5D=u8CcwtuWSyw1r2s4BeYRthBriYej6bKREffVvdoYpuQNHfdh64FPPqp2JSPXFZwwlUj60bfsyR2SMkeg2-YvA6vaHQ.X_L0q3__3nq-Mbl_tAoq4-cXXycZyhX90DuPB2a344IkBNxCuiwOrv4uTa2ji7OUM68HnlEuv9oYFK-gE6yBww).

6. Maki BE, Holliday PJ, Topper AK. A prospective study of postural balance and risk of falling in an ambulatory and independent elderly population. J Gerontol, 1994;49(2):M72-84. doi:10.1093/geronj/49.2.m72.

**FIGURES**

**
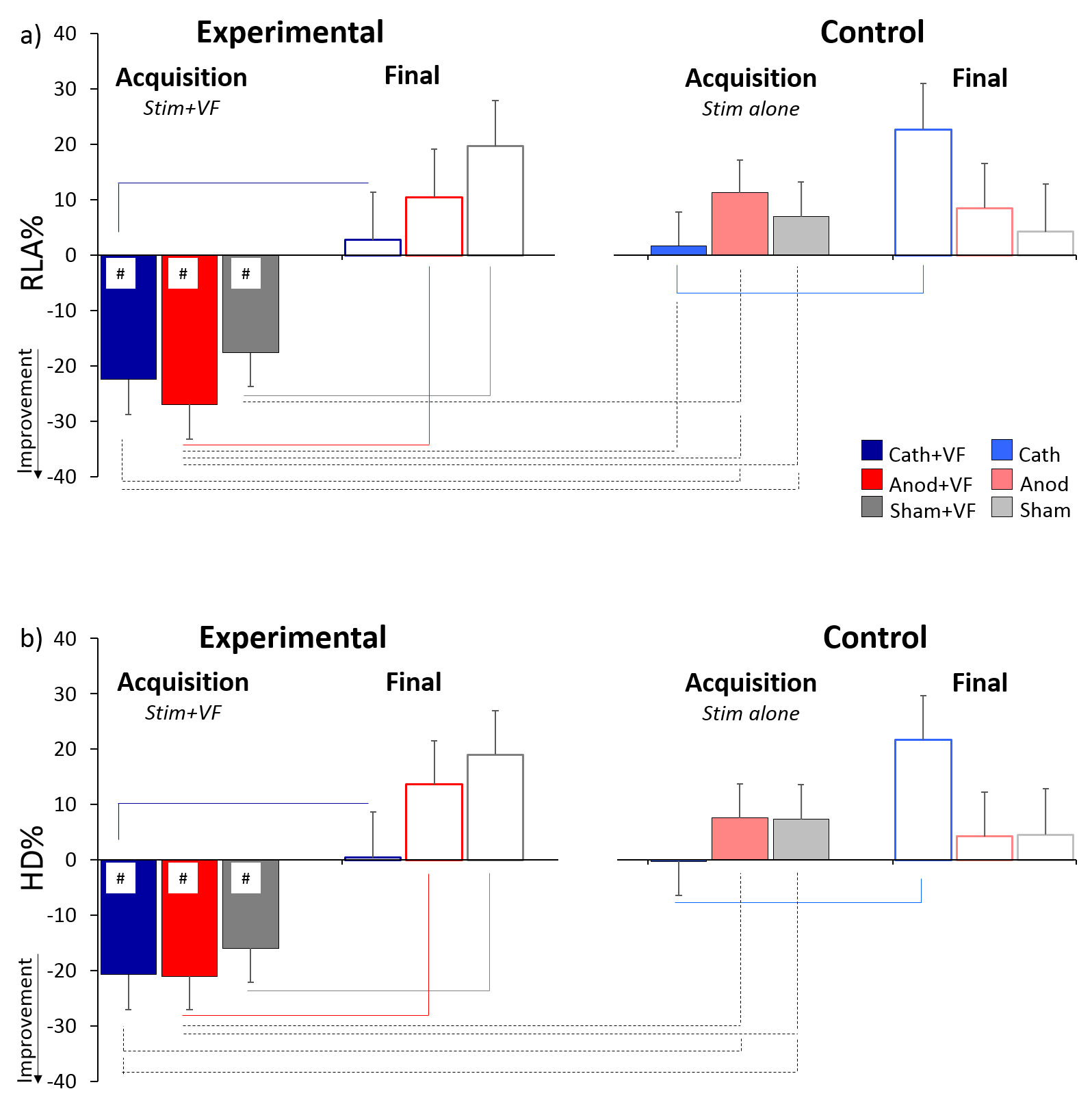
**

**Fig. S1** Body sway in the anteroposterior direction. **a**) RLA% and **b**) HD% were more reduced during the acquisition (full bars) than during the final session (empty bars) for the experimental groups (left panel) provided with visual feedback. The # denotes significant difference from zero. When visual feedback was provided, the values were significantly lower than zero during the acquisition session for the cathodal (blue full bar), the anodal (red full bar), and the sham (grey full bar) group. Horizontal lines (solid and dashed) indicate significant differences between groups. Significance level (p < 0.05)


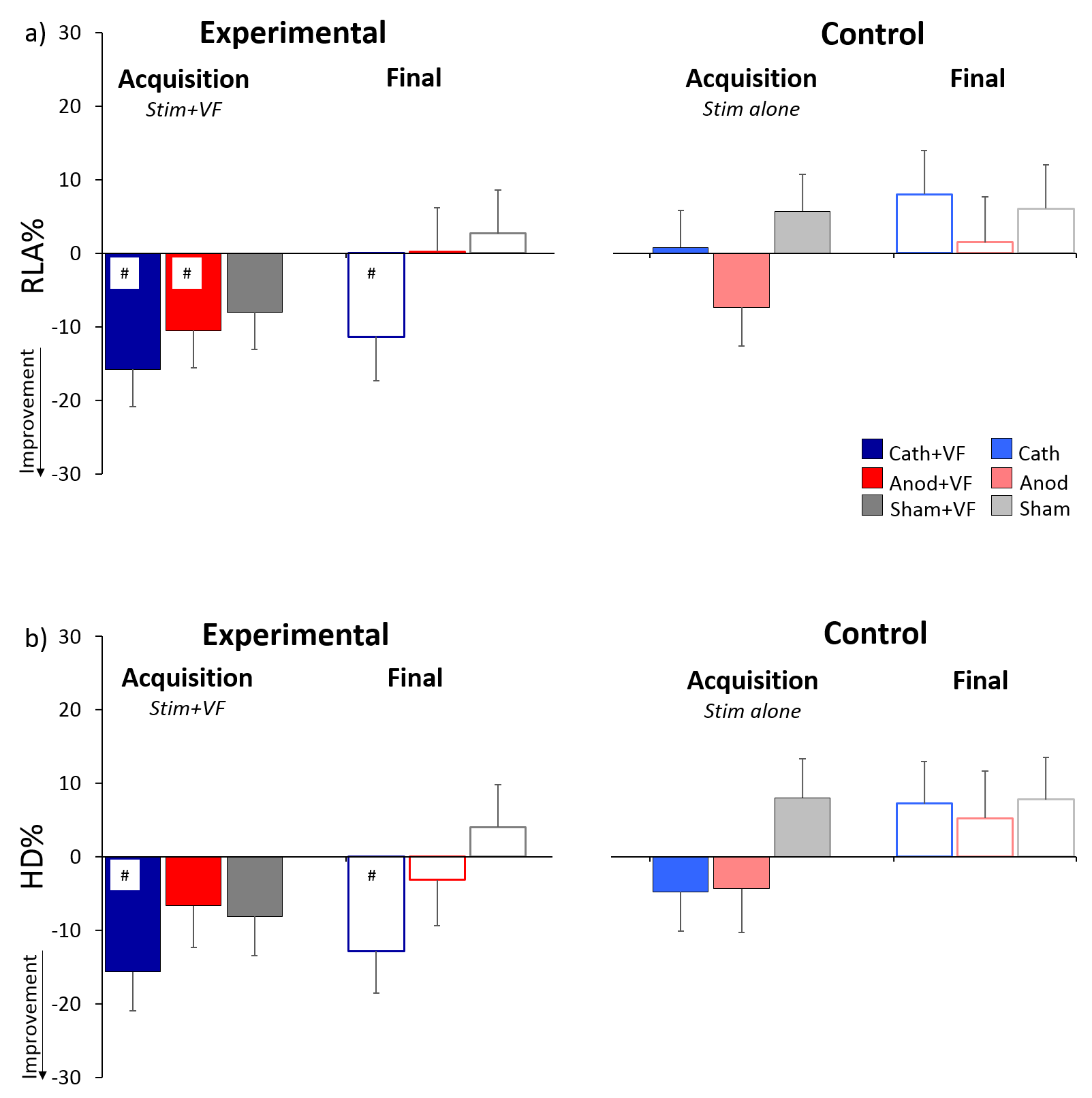


**Fig. S2** Body sway in the mediolateral direction. **a**) RLA% was significantly lower than zero (#) during the acquisition session (full bars) for the cathodal and the anodal group provided with visual feedback (left panel). **b**) HD% was significantly lower than zero only in the cathodal group provided with visual feedback (left panel). In both cases, the values for the cathodal group provided with visual feedback (empty bar with blue border) remained lower than zero also during the final session, after the removal of visual feedback. Significance level (p < 0.05)

**Supplementary Table 1.** Percentage of outlier values and number of outlier subjects in each group and in each index.

|  | | **Groups** | | | | | | | | | | | |
| --- | --- | --- | --- | --- | --- | --- | --- | --- | --- | --- | --- | --- | --- |
|  |  | **Cath+VF** | | **Anod+VF** | | **Sham+VF** | | **Cath** | | **Anod** | | **Sham** | |
|  |  | Outlier  values | Outlier  subjects | Outlier  values | Outlier  subjects | Outlier  values | Outlier  subjects | Outlier  values | Outlier  subjects | Outlier  values | Outlier  subjects | Outlier  values | Outlier  subjects |
| Maximal | RLA | 5.56% | 1 | 5.78% | 1 | 4.44% | 1 | 6.22% | 2 | 6.44% | 1 | 6.44% | 2 |
|  | HD | 5.78% | 3 | 5.56% | 1 | 4.89% | 1 | 6.89% | 2 | 6.22% | 1 | 6.22% | 2 |
| Anteroposterior | RLA | 5.78% | 2 | 6.00% | 2 | 5.56% | 1 | 7.11% | 1 | 6.00% | 0 | 5.33% | 2 |
|  | HD | 5.56% | 2 | 6.22% | 1 | 5.56% | 1 | 7.33% | 1 | 5.56% | 1 | 5.78% | 2 |
| Mediolateral | RLA | 4.44% | 0 | 4.89% | 0 | 4.44% | 0 | 5.11% | 0 | 4.89% | 1 | 5.33% | 0 |
|  | HD | 4.89% | 0 | 4.89% | 2 | 4.44% | 0 | 6.00% | 0 | 5.56% | 3 | 5.56% | 1 |

RLA = relative leg angle; HD = hip displacement.

**Supplementary Table 2**. Normalized mean values and standard error (±) in the acquisition and final sessions for the six groups.

|  | | **Groups** | | | | | | | | | | | |
| --- | --- | --- | --- | --- | --- | --- | --- | --- | --- | --- | --- | --- | --- |
|  |  | **Cath+VF** | | **Anod+VF** | | **Sham+VF** | | **Cath** | | **Anod** | | **Sham** | |
|  |  | Acquisition | Final | Acquisition | Final | Acquisition | Final | Acquisition | Final | Acquisition | Final | Acquisition | Final |
| Maximal | RLA | -23.1±3.2 | -8.1±3.5 | -16.2±3.7 | 8.8±6.6 | -10.4±4.7 | 15.7±6.3 | -10.2±5.8 | 7.0±8.4 | 10.4±5.9 | 8.6±5.5 | 5.3±6.4 | 6.5±5.9 |
|  | HD | -21.7±2.7 | -8.4±3.1 | -15.9±3.9 | 9.9±6.5 | -13.2±4.1 | 16.5±6.5 | -12.3±5.9 | 7.5±8.5 | 9.6±6.6 | 8.0±5.8 | 0.9±8.5 | 3.7±6.1 |
| Anteroposterior | RLA | -22.5±3.3 | 2.8±5.2 | -27.0±3.7 | 10.5±6.1 | -17.7±3.7 | 19.7±10.0 | 1.7±8.8 | 22.7±12.9 | 11.3±5.9 | 8.5±5.9 | 7.0±8.7 | 4.2±7.3 |
|  | HD | -20.7±2.6 | 0.4±4.0 | -21.0±3.9 | 13.6±7.0 | -16.0±4.2 | 19.0±9.3 | -0.3±7.9 | 21.7±12.0 | 7.6±6.8 | 4.3±6.6 | 7.3±9.1 | 4.6±6.2 |
| Mediolateral | RLA | -15.8±3.7 | -11.4±4.7 | -10.5±4.8 | 0.2±6.3 | -8.0±5.1 | 2.7±4.6 | 0.8±7.6 | 8.1±7.5 | -7.3±4.0 | 1.5±7.3 | 5.7±4.3 | 6.1±5.2 |
|  | HD | -15.6±2.9 | -12.8±4.7 | -6.7±5.0 | -3.1±3.8 | -8.1±4.2 | 4.0±4.7 | -4.8±6.4 | 7.3±7.5 | -4.4±4.3 | 5.2±7.6 | 8.1±7.9 | 7.8±6.2 |

RLA = relative leg angle; HD = hip displacement.
